# Clinical Comparison of Ketamine‐Dexmedetomidine With Ketamine‐Propofol During Canine Orchiectomy: A Randomized Study

**DOI:** 10.1002/vms3.70412

**Published:** 2025-05-26

**Authors:** Mumin Gokhan Senocak

**Affiliations:** ^1^ Department of Surgery, Faculty of Veterinary Medicine Atatürk University Erzurum Turkey

**Keywords:** canine castration, dexmedetomidine, ketamine, ketadex, ketofol, propofol

## Abstract

**Objective:**

To compare the effects of two different protocols, ketamine with dexmedetomidine (ketamine‐dexmedetomidine [KD]) and ketamine with propofol (ketamine‐propofol [KP]), on intubation time, selected cardiopulmonary parameters, and anaesthesia maintenance during canine orchiectomy in a clinical setting.

**Study Design:**

Randomized clinical trial.

**Animals:**

Twenty‐six healthy dogs were undergoing orchiectomy.

**Methods:**

Dogs were randomly assigned to the KD group [*n* = 13; single intravenous bolus of ketamine (5 mg kg^−1^) combined with dexmedetomidine (10 µg kg^−1^)], or KP group [*n* = 13, an intravenous bolus of ketamine combined with propofol at a 1:2 concentration ratio and infused at a 0.2 mL kg^−1^ min^−1^ rate for 120 s until jaw relaxation and the consumed amount recorded]. Orotracheal intubation followed the induction of anaesthesia. The cardiopulmonary variables were assessed at baseline and 5‐min intervals up to 30 min. A 20% increase in at least two variables, such as heart rate (HR), mean arterial pressure (MAP) and respiratory rate, prompted the administration of top‐ups. Following surgery, the recovery time and quality were assessed.

**Results:**

There was no significant difference in intubation time between KD (3.3 ± 0.8) and KP (2.7 ± 0.9, *p* = 0.121). Over time, HR and MAP significantly increased in the KP group compared to the KD group (*p* < 0.001). The haemoglobin oxygen saturation was higher in the KD group (97.7% ± 2.1%) compared to the KP (95.3% ± 2.2%, *p* = 0.015). The duration of the top‐up requirement was longer in the KD group as compared to a single bolus of KP, with a mean difference of 31.2 min (95% CI 20.80–41.51) (*p* < 0.01).

**Conclusions and Clinical Relevance:**

Both KP and KD combinations effectively maintain anaesthesia during canine castration surgery, demonstrating comparable intubation times. Although KP requires additional top‐ups, it potentially offers enhanced cardiovascular stability compared to KD. However, the use of KP necessitates support of body temperature and oxygenation.

## Graphical abstract

The graphical abstract of this study presents a comparative analysis of two anaesthetic protocols, ketamine‐dexmedetomidine and ketamine‐propofol, utilized during canine orchiectomy.

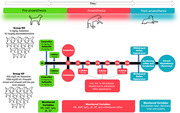

## 1 Introduction

Injectable anaesthetics ketamine and propofol are often utilized to facilitate intubation in dogs (Riccó and Henao‐Guerrero 2014). Including ketamine in the anaesthetic protocol lengthens the intubation period, and the delivery route may alter intubation and induction of anaesthesia (White et al. 2001).

The *N*‐methyl‐d‐aspartate receptor antagonist ketamine can induce and maintain anaesthesia (Kennedy and Smith 2015). It induces a state of dissociative anaesthesia and reduces nociception tone. Ketamine is a sympathetic activity stimulant that may offset α2‐adrenoceptor agonist‐induced bradycardia (Haskins et al. 1985). In order to produce anaesthesia in dogs, ketamine is often combined with propofol or dexmedetomidine (Barletta et al. 2011; Martinez‐Taboada and Leece 2014).

Dexmedetomidine is the most selective α2‐adrenoceptor agonist for predictable and reliable sedation, muscle relaxation and analgesia in dogs. (Raekallio et al. 2000; Leppänen et al. 2006; Paddleford and Harvey 1999). As a premedication agent, dexmedetomidine has an anaesthetic‐sparing effect on the required injectable anaesthetics. By activating α2‐adrenoceptors in the central nervous system, particularly in the locus coeruleus, it inhibits norepinephrine release, reducing sympathetic nervous system activity. This mechanism decreases the requirement for other anaesthetic agents to achieve and maintain the desired depth of anaesthesia (Gómez‐Villamandos et al. 2006). The cardiovascular effects of dexmedetomidine administration in dogs most frequently include decreased cardiac output, bradyarrhythmia and peripheral vasoconstriction (Bloor et al. 1992).

Propofol, a commonly used sedative‐hypnotic for anaesthesia induction (Sano et al. 2003; Dogan et al. 2016; Yanmaz et al. 2020), can cause significant cardiac and neurological depression in dogs (Whitwam et al. 2000). Combining it with ketamine, known as ‘ketofol’, enhances cardiovascular stability and reduces anaesthetic consumption by lowering the required doses (Shinde et al. 2018). In human medicine, ketamine combined with dexmedetomidine or propofol is frequently used and considered equivalent (Tekeli et al. 2020; Canpolat et al. 2017; Amer et al. 2020). Recent studies have investigated these combinations for deep sedation and analgesia in paediatric procedures like transcatheter atrial septal defect closure, burn dressing changes and tooth extraction (Canpolat et al. 2017, 2012; Koruk et al. 2010).

Despite the frequent usage of both combinations in dogs, no comparative study has been conducted in veterinary practice. Although ketamine and propofol are often co‐administered intravenously, there is a lack of clinical data on the efficacy of combining ketamine with dexmedetomidine and administering them as a single intravenous bolus for the induction of anaesthesia in dogs.

This study aims to test the following null hypotheses: There is no significant difference in terms of intubation time, heart rate (HR), indirect mean arterial pressure (MAP), peripheral oxygen saturation, respiratory rate or rectal temperature (RT) between two clinical protocols, ketamine‐dexmedetomidine (KD) (a single bolus of 5 mg kg^−1^ ketamine and 10 µg kg^−1^ dexmedetomidine) and ketamine‐propofol (KP) (a mixture of ketamine and propofol in the same syringe at a 1:2 ratio of milligrams, administered intravenously at a rate of 0.2 mL kg⁻^1^ min⁻^1^ for 120 s until jaw tone relaxation is achieved), during canine orchiectomy. Additionally, the study posits that maintaining anaesthesia during orchiectomy with these induction agents is not feasible.

## 2 Materials and Methods

The study was conducted in a spay and neuter program of a shelter in a veterinary teaching hospital with the approval of the Atatürk University Local Boards of Ethics Committee (2022/6‐99 and 2022/10‐198). All procedures were carried out in an accredited surgery theatre by board‐certified specialists.

### 2.1 Animals

A total of 26 crossbreed healthy male dogs were used for the study. The study's inclusion criteria included dogs with a friendly disposition towards humans, as indicated by their positive response to wagging their tails when approached. A physical examination was performed to determine the health state of the animal. Inclusion criteria were an American Society of Anesthesiologists (ASA) physical classification status score of I, a complete blood count with no values outside the reference interval, a body condition score of four or five out of nine, and the absence of a history of systemic disease.

Exclusion criteria were dogs exhibiting aggressive behaviour or an indirect MAP with readings below 60 mmHg during baseline (BL) data recording.

### 2.2 Study Design

The study was a prospective, double‐blinded, randomized clinical trial.

The dogs were fasted for a minimum of 6 h with water available until 30 min prior to anaesthesia. Two hours before the surgery, a subcutaneous injection of carprofen at a dosage of 4.4 mg kg^−1^ (Rimadyl, Zoetis, Türkiye) was administered. Dogs were transported to the preoperative preparation room, and the right cephalic vein was cannulated with a catheter (20 Ga, 1.25 in, Ayset, Türkiye). A single cefazolin 22 mg kg^−1^ (Rimadyl, Türkiye) dose was administered intravenously. The dogs were transferred to the surgery room after the surgical sites were clipped and aseptically prepared.

### 2.3 Randomization

A technician unaware of the treatment protocols randomly assigned dogs to the KD or KP group through a randomized drawing of lots.

### 2.4 Induction

The KD group (*n* = 13), received a single intravenous bolus of 5 mg kg^−1^ ketamine (Ketasol 100 mg mL^−1^, Interhas, Richter Pharma AG, Wels, Austria) and 10 µg kg^−1^ dexmedetomidine (Dekstomid, 100 µg mL^−1^, Polifarma, Tekirdag, Turkey) in the same syringe. The KP group (*n* = 13) received a mixture of ketamine and propofol (B Braun Ltd, 10 mg mL^−1^, Melsungen, Germany) in the same syringe at a 1:2 ratio of milligrams (Şenocak 2023). Preparing the KP combination involved combining 0.5 mL of ketamine and 10 mL of propofol. The KP bolus was administered intravenously at a rate of 0.2 mL kg^−1^ min^−1^ for 120 s until the jaw tone relaxed.

### 2.5 Intubation and Ventilation

In order to determine the appropriate length for the endotracheal tube, the distance from the tip of the dog's nose to the point of the shoulder was measured. The diameter of the tube was determined by dividing the width of the dog's nose by three. The trachea was intubated (T0) with an endotracheal tube, and the dog was in sternal recumbency. The cuff was inflated, and the minimal occluding volume was tested using a stethoscope from the ventral midline of the mid‐neck. The tube was then secured to the lower jaw with gauze bandage material, and the dog was positioned in left lateral recumbency. The tube was attached to a circle breathing system, delivering a flow of 2 L min^−1^ of 100% oxygen. Manual ventilation was administered using a reservoir bag if the patient monitor's haemoglobin oxygen saturation (SpO_2_) level fell below 92%.

### 2.6 Maintenance of Anaesthesia

Anaesthesia was maintained by administering additional top‐ups equivalent to the initial bolus. A 20% increase in two out of three variables, such as MAP, HR or respiratory rate (*f*_R_), determined the timing of these additional doses.

### 2.7 Measurements

A multiparameter (MVM Medical, GT9000F, Türkiye) patient monitor was utilized to record the MAP (mmHg), HR (beats min^−1^), *f*_R_ (breaths minute^−1^), SpO_2_ (%) and RT (°C). The variables, including the MAP, HR, *f*_R_, SpO_2_, RT and withdrawal reflex, were recorded at BL, T0 and 5‐min intervals for 30 min (T5, T10, T15, T20, T25 and T30).

BL data was collected 3 min after positioning the dogs in the left lateral recumbency to allow them to acclimate to their surroundings and achieve a state of relaxation. The SpO_2_ infrared probe was initially placed on the ear to record BL data and moved to the tongue after induction of anaesthesia to obtain measurements of HR and SpO_2_. ECG leads were attached to the forelimbs and left hindlimb to measure HR and *f*_R_. A blood pressure cuff measuring the indirect MAP with a diameter of about 40% of the limb circumference was placed immediately proximal to the tarsus on the right pelvic limb. A rectal probe was lubricated and inserted approximately 1.5 cm into the rectum to measure RT.

During the study, a contingency plan was established for hypotension (MAP < 60 mmHg) involving intravenous ephedrine at 0.1 mg kg^−1^.

### 2.8 Surgery

The surgical site was prepared with the administration of the initial bolus, and the practitioner carried out the preparation. The surgical procedure started 5 min after the intubation and was performed with the patient in left lateral recumbency. The right leg was raised and secured to the surgical table using gauze bandage material. An orchiectomy was performed through a single incision in the pre‐scrotal region, and the incision was sutured. The surgery time was recorded. Even if the surgery had been completed, anaesthesia was administered until the 30‐min threshold.

### 2.9 Extubation and Recovery

Following surgery, the trachea was extubated once the swallowing reflex had returned. Extubation time was recorded, and dogs were transferred to a pre‐warmed (between 20°C and 24°C) recovery room via an anaesthesia trolley and placed on the floor bed in left lateral recumbency. The door was closed, and the practitioner assessed the quality of recovery and recorded recovery time by observing the dogs through the recovery room window. The recovery quality was assessed on a five‐point scale: 1, very smooth; 2, fairly smooth; 3, moderately smooth; 4, not smooth; and 5, severe agitation with hostility (Lozano et al. 2009). The dogs were deemed to have recovered once they returned spontaneously to a sternal position without external stimulation.

The administration of infusions and the recording of the amounts consumed were performed by Student 1, who was aware of the treatments and was also responsible for infusing the drug accordingly on the basis of the feedback received from Student 2 or practitioner. Student 2 assessed jaw relaxation and withdrawal reflex and recorded MAP, HR, *f*_R_, SpO_2_ and RT variables but was unaware of treatments.

The practitioner, who was responsible for conducting the surgery, evaluating the cardiorespiratory variables during surgery, giving feedback to Student 1 to infuse additional top‐ups and scoring the recovery, was unaware of the treatments.

### 2.10 Statistical Analysis

Statistical analyses were performed using SPSS software (Version 22, IBM Corp., Armonk, NY, USA). The normality of data was tested with the Shapiro–Wilk test. The homogeneity of the variances was tested using Levene's test.

Sample size calculation (MedCalc Software Ltd, V20.015) was determined on the basis of the time to achieve intubation as the study's primary outcome. As there were no previous comparative studies between KD and KP in veterinary medicine, the sample size was determined by identifying the differences in intubation times from a similar study (El Mourad et al. 2019). This calculation revealed that 13 animals per group were required to detect an 8.15‐min difference between regimens, as reported in a previous study. As only the intubation time was considered the primary outcome in calculating the sample size for the study, observed power percentages were presented to comprehend the effect size of statistically significant data obtained from other variables.

The independent samples *t*‐test was employed to compare the durations of intubation, extubation, recovery, surgery and the requirement for top‐up. The quality of recovery was compared among groups using the Mann–Whitney *U*‐test. Additionally, a multivariate ANOVA was used to compare bolus times and the consumed amounts of ketamine and propofol between boluses, with pairwise comparisons adjusted using a Bonferroni correction.

Data of MAP, HR, *f*_R_, SpO_2_ and RT variables were compared among time points (BL, T0, T5, T10, T15, T20, T25 and T30) using repeated measures of one‐way ANOVA and pairwise comparisons adjusted using a Bonferroni correction. If sphericity could not be assumed, a Huynh–Feldt correction was applied when the epsilon was greater than 0.75, and a Greenhouse–Geisser correction was used if it was less than or equal to 0.75. The variables between groups were compared using a linear mixed model. Adjustments for multiple comparisons were made with Bonferroni correction.

The parametric data are presented as mean ± standard deviation. Mean differences between combinations are presented as mean differences (95% CI lower bound–upper bound). The recovery quality is given as the median rank and range. A level of alpha (Type I error) 0.05 is considered statistically significant.

## 3 Results

The study initially intended to use 28 healthy male crossbred dogs. However, two dogs were deemed unsteady MAP variables and were not eligible for the study due to inconsistent MAP readings (below 60 mmHg) with patient monitor during the BL recording conducted before anaesthesia administration. As a result, the surgical procedures for these two dogs were postponed, and they were referred to the internal medicine clinic for further evaluation.

**FIGURE 1:**
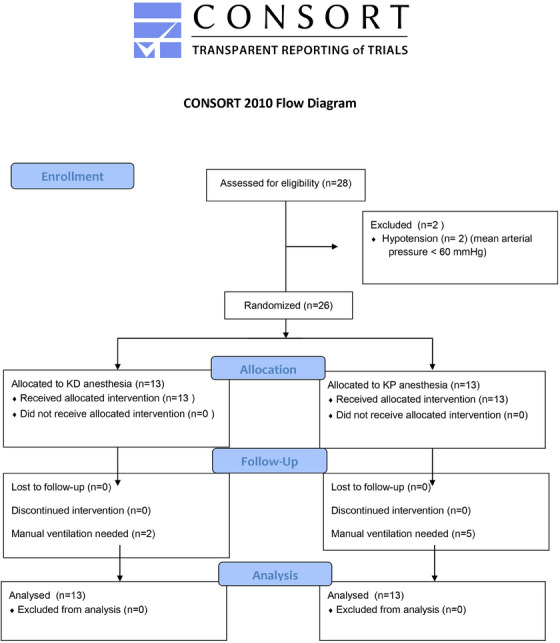
CONSORT flow diagram illustrating the process of enrolment, allocation, follow‐up and analysis conducted in the study.

A total of 26 crossbreed healthy male dogs were used for the study (Figure 1). No transient adverse effects, such as tremors, vomiting, sneezing, lacrimation, salivation, excitement, stiffness, nausea or hypotension, were observed in either group.

Due to a drop of SpO_2_ below 92%, manual ventilation was required in seven dogs, two in the KD and five in the KP group.

In the KD group, manual ventilation was administered to two dogs for 7 and 9 min, respectively. This intervention was initiated 8 min following the initial bolus. In the KP group, manual ventilation was required for the first bolus infusion in one dog (dog a) for 2 min, for the second bolus infusion in three dogs (dogs a–c) for 3 min, for the third bolus infusion in three dogs (dogs a, b and d) for 3–4 min, and for the fourth bolus infusion in two dogs (dogs a and b) for 4 min.

The duration required to achieve endotracheal intubation did not exhibit a significant difference between the KD and KP groups, with a mean difference of 0.6 min (95% CI: −0.15–1.23, *p* = 0.121). The surgery time did not differ significantly between the groups (*p* = 0.086) (Table 1).

**TABLE 1:** Comparison of KD and KP combinations in dogs.

Parameter	KD (*n* = 13)	KP (*n* = 13)	Observed power (%)	*p*
Age (years)	6.5 ± 4.9	6.1 ± 3.6	6	0.817
Body weight (kg)	29.4 ± 9.1	33.5 ± 4.1	33	0.179
Time to achieve intubation (minute)	3.3 ± 0.8	2.7 ± 0.9	34	0.121
Duration to top‐up requirement (minute)	39.4 ± 19.3	8.2 ± 2.9	100	<0.01
Extubation time (minute)	45.2 ± 19.2	35.2 ± 7.8^a^	41	0.129
Recovery time (minute)	52.8 ± 33.3	50 ± 20^a^	6	0.797
Recovery quality (score)	1 (1–3)	1 (1–3)	10	0.421
Surgery time (minute)	13.09 ± 1.64	14.26 ± 1.67	40	0.086

*Note*: The collected data includes age (years), body weight (kg), time taken to achieve intubation (minutes), duration to top‐up requirement (minutes), extubation time (minutes), recovery time (minutes), recovery quality (score) and surgery time (minutes). Parametric data are presented as mean ± standard deviation, whereas nonparametric data are reported as median (range). The ‘*n*’ represents the number of animals included in the study. In the KD (ketamine‐dexmedetomidine) combination, 5 mg kg^−1^ of ketamine was combined with 10 µg kg^−1^ of dexmedetomidine in the same syringe and administered as a bolus infusion. On the other hand, the KP (ketamine‐propofol) combination refers to a mixture of 1/2 ketamine (500 mg in 5 mL) and propofol (1000 mg in 100 mL). The KP combination was infused until the jaw tone relaxed.

^a^ The reported extubation and recovery times in the KP combination pertain to the results obtained after multiple‐bolus administration.

The duration of requiring a top‐up was longer in the KD group compared to the KP group, with a mean difference of 31.2 min (95% CI 20.80–41.51, *p* < 0.01) (Table 1).

No difference was observed in extubation time, recovery time and recovery quality between the KD and KP groups (Table 1).

In the KP group, the ketamine and propofol required to achieve endotracheal intubation were 1.9 ± 0.3 and 3.9 ± 0.6 mg kg^−1^, respectively (Table 2).

**TABLE 2:** Mean consumed amounts of ketamine and propofol in ketamine‐propofol (KP) combination and mean time between boluses.

Bolus	Ketamine (mg kg^−1^)	Propofol (mg kg^−1^)	*p*
First bolus	1.9 ± 0.3	3.9 ± 0.6	0.004^a^, < 0.001^b^, < 0.001^c^, 0.037^d^, <0.001^e^, 0.001^f^
Time between boluses (minute)	8.2 ± 2.9		
Second bolus	1.4 ± 0.4	2.7 ± 0.8	
Time between boluses (minute)	11.8 ± 4.6		
Third bolus	1.09 ± 0.3	2.17 ± 0.6	
Time between boluses (minute)	11.5 ± 0.6		
Fourth bolus	0.6 ± 0.1	1.1 ± 0.1	

*Note*: Data are reported as the mean ± standard deviation. In the KP combination, which refers to a mixture of 1/2 ketamine (500 mg in 5 mL) and propofol (1000 mg in 100 mL), the infusion is continued until the jaw tone relaxation. The following differences are noted.

^a^ Difference between the first and second boluses.

^b^ Difference between the first and the third bolus.

^c^ Difference between the first and the third bolus.

^d^ Difference between the second and the third bolus.

^e^ Difference between the second and the fourth bolus.

^f^ Difference between the third and the fourth bolus.

In the KP group, additional top‐ups were required to maintain anaesthesia throughout the surgery. Conversely, in the KD group, a single dose infusion was administered solely, and there was no necessity for additional top‐ups. The mean induction doses for ketamine and propofol were higher than those of the second bolus, with a mean difference of 0.56 mg kg^−1^ ketamine (95% CI: 0.30–0.82) and 0.13 mg kg^−1^ propofol (95% CI: 0.60–1.64), respectively (*p* < 0.01). The mean dose of the second bolus of ketamine and propofol was higher than that of the third bolus, with a mean difference of 0.28 mg kg^−1^ ketamine (95% CI: 0.02–0.54) and 0.57 mg kg^−1^ propofol (95% CI: 0.04–1.09), respectively (*p* = 0.036). The mean dose of the third bolus of ketamine and propofol was higher than that of the fourth bolus, with a mean difference of 0.53 mg kg^−1^ ketamine (95% CI: 0.35–0.71) and 1.07 mg kg^−1^ propofol (95% CI: 0.71–1.42), respectively (*p* < 0.01), (Table 2). All dogs required a second bolus, 12 animals required a third one, and only 2 needed a fourth one to maintain the 30‐min duration.

Within the KP group, the mean time interval between the second and third boluses was significantly greater (*p* = 0.009) than between the first and second boluses, with a mean difference of 3.63 min (95% CI: 0.75–6.50). Similarly, the mean time interval between the third and fourth boluses was significantly greater (*p* = 0.020) than that between the first and second boluses, with a mean difference of 3.30 min (95% CI: 0.42–6.17) (Table 2).

**FIGURE 2:**
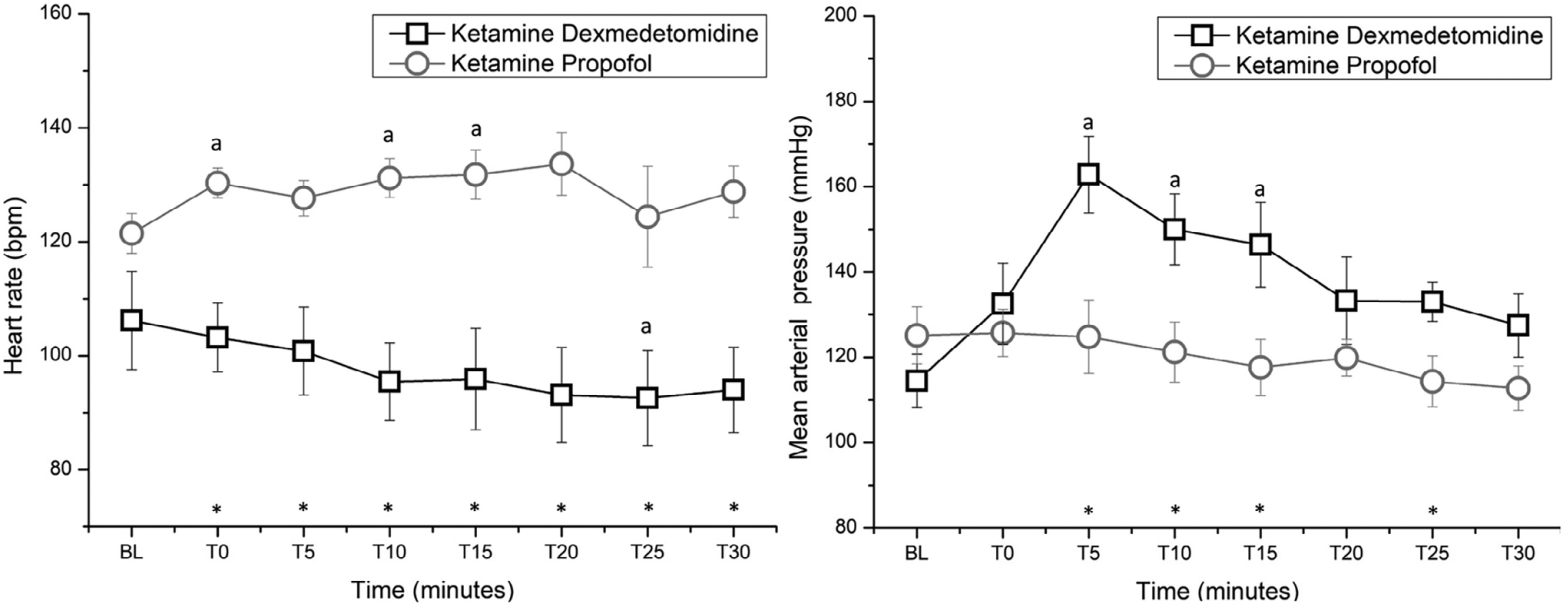
Physiological variables over time in dogs receiving ketamine combined with propofol (KP) or dexmedetomidine (KD). The heart rate and indirect mean arterial blood pressure were measured at baseline (BL), induction time (T0), as well as at T5, T10, T15 and T30 minutes thereafter. In the KD combination, 5 mg kg^−1^ of ketamine was combined with 10 µg  kg^−1^ of dexmedetomidine in the same syringe and administered as a bolus infusion. The KP combination involved a mixture of 1/2 ketamine (500 mg in 5 mL) and propofol (1000 mg in 50 mL), which was infused until the jaw tone relaxed. Significant differences from the BL are denoted as ‘a’ (*p* < 0.05). Additionally, significant differences were observed between the KD and KP combination groups, denoted by an asterisk (*p* < 0.05).

The HR was significantly increased at T0 (observed power: 100%, *p* < 0.01), T10 (observed power: 80%, *p* = 0.009) and T15 (observed power: 70%, *p* = 0.018) in the KP group when compared to BL. In the KD group, HR significantly decreased at T25 (observed power: 53%, *p* = 0.047) (Figure 2).

The HR was significantly higher in the KP combination than in the KD combination at T0 (*p* < 0.001), T5 (*p* = 0.002), T10 (*p* < 0.001), T15 (*p* = 0.001), T20 (*p* < 0.001), T25 (*p* = 0.018) and T30 (*p* < 0.001), with mean differences of 27 bpm (95% CI 13.02–41.28), 26 bpm (95% CI 11.33–42.37), 35 bpm (95% CI 21.23–50.25), 35 bpm (95% CI 14.74–57.04), 40 bpm (95% CI 20.77–60.38), 32 bpm (95% CI 5.88–57.83) and 35 bpm (95% CI 17.69–51.91), respectively (Figure 2).

The MAP in the KD group increased at T5 (observed power: 93%, *p* = 0.003), T10 (observed power: 97%, *p* = 0.001) and T15 (observed power: 72%, *p* = 0.018) compared to BL. However, there was no significant difference in MAP over time in the KP group (*p* = 0.47) (Figure 2).

In the KD group, the MAP was significantly higher compared to the KP group at T5 (*p* = 0.006), T10 (*p* < 0.001), T15 (*p* = 0.019) and T25 (*p* = 0.028). The mean differences in MAP between the KD and KP groups were 38 mmHg (95% CI 11.84–64.19), 30 mmHg (95% CI 6.33–51.27), 29 mmHg (95% CI 5.08–52.44) and 19 mmHg (95% CI 2.13–35.19) at T5, T10, T15 and T25, respectively (Figure 2).

The SpO_2_ did not significantly change in the KP (*p* = 0.449) and KD (*p* = 0.77) groups over time. However, SpO_2_ was significantly higher in the KD group at T10 (*p* = 0.009), with a mean difference of 2.5% (95% CI 0.67–4.25) compared to the KP group (Table 3).

**TABLE 3:** Physiological variables over time in dogs receiving ketamine combined with propofol (KP) or dexmedetomidine (KD).

Variable	Combination	Time points							
		BL	T0	T5	T10	T15	T20	T25	T30
SpO_2_ (%)	**KD**	96.6 ± 3.1	95.7 ± 3.4	95.9 ± 3.2	**97.7** ± 2.1*	95.8 ± 3	96.4 ± 3.6	96 ± 3.2	96.5 ± 3
	**KP**	96.1 ± 2.1	94.7 ± 2.8	94.6 ± 2.3	**95.3** ± 2.2*	96 ± 2.4	95.3 ± 2.3	95.5 ± 2.1	96 ± 2.2
*f*_R_ (breath per minute)	**KD**	20 ± 6	20 ± 5	**21** ± 5*	20 ± 5	20 ± 5	17 ± 2	20 ± 6	20 ± 5
	**KP**	20 ± 5	**17** ± **3^a^**	**17** ± 4*^a^	20 ± 2	18 ± 3	18 ± 4	**17** ± **3^a^**	18 ± 3
RT (°C)	**KD**	37.9 ± 3.1	38.8 ± 0.4	38.9 ± 0.4	38.9 ± 0.3	38.9 ± 0.3	38.9 ± 0.3	38.9 ± 0.4	38.8 ± 0.4
	**KP**	38.9 ± 0.5	38.8 ± 0.6	38.7 ± 0.7	38.7 ± 0.7	**38.7** ± **0.7^a^**	**38.7** ± **0.7^a^**	**38.6** ± **0.9^a^**	**38.5** ± **0.9^a^**

*Note*: Peripheral haemoglobin oxygen saturation (SpO_2_), respiratory frequency (*f*_R_) and rectal temperature (RT) were measured at baseline and at specific time points: Induction time, T5, T10, T15 and T30 minutes after T0. In the KD combination, 5 mg kg^−1^ of ketamine was combined with 10 µg kg^−1^ of dexmedetomidine in the same syringe and administered as a bolus infusion. The KP combination involved a mixture of 1/2 ketamine (500 mg in 5 mL) and propofol (1000 mg in 100 mL), which was infused until the jaw tone relaxed. The measurements were taken at various time points: baseline (BL), T0 (induction time), T5, T10, T15 and T30 minutes after T0. The data are presented as mean ± standard deviation. Significant differences from the baseline (BL) are denoted as ‘a’ (*p* < 0.05). Additionally, significant differences were observed between the KD and KP combination groups, denoted by an asterisk (*p* < 0.05). Statistically significant differences are marked in bold.

The *f*_R_ in the KP group was significantly increased at T0 (observed power: 53%, *p* = 0.046), T5 (observed power: 53%, *p* = 0.047) and T25 (observed power: 61%, *p* = 0.03) when compared to BL. In the KD group, *f*_R_ did not change significantly over time (*p* = 0.393). The *f*_R_ was significantly higher in the KD group at T5 (*p* = 0.043), with a mean difference of four breaths per minute (95% CI 0.12–7.18), compared to the KP group (Table 3).

The RT was significantly decreased at T15 (observed power: 53%, *p* = 0.04), T20 (observed power: 69%, *p* = 0.019), T25 (observed power: 68%, *p* = 0.02) and T30 (observed power: 79%, *p* = 0.01) compared to BL over time in the KP group. However, RT was not significantly changed over time compared to BL in the KD group. The RT was not significantly different between groups over time (*p* = 0.637, Table 3).

Notably, all dogs in the KP group showed an unknown stretching phenomenon a few times with their hind legs during recovery.

## 4 Discussion

Both the KP and the KD are effective injectable anaesthetic protocols for healthy dogs undergoing orchiectomy without premedication. Although intubation time did not differ between the two, lower MAP, SpO_2_ and *f*_R_ but higher HR were observed with KP compared to KD.

Although KD and KP are used in human and veterinary medicine due to their profound sedative and analgesic effects (Canpolat et al. 2017, 2012; Koruk et al. 2010), there is no clear information on how these protocols should be used for anaesthesia induction with maintenance or for the timing of additional dog doses.

Insufficient anaesthetic level fosters instability in haemodynamic variables such as HR, MAP and *f*_R_. Some literature suggests that surgeons, during surgery, assess withdrawal reflex for anaesthesia level (Iqbal et al. 2020; Cardoso et al. 2014), which occasionally signifies the timing for additional top‐ups. Certain researchers employ a criterion of a 20% elevation in two of the three variables, namely, HR, MAP and *f*_R_, during surgery to identify the appropriate time for top‐ups (Şenocak 2023; Mansour et al. 2017). The results of this study demonstrated that both KD and KP provided adequate anaesthesia in dogs undergoing orchiectomy. However, although a single dose of KD sufficed for surgical plane anaesthesia, additional KP top‐ups were required after approximately 8 min.

The results of a study on dogs indicate that intubation was only possible after 10 min following the intramuscular administration of the KD combination (Barletta et al. 2011). However, in the present study, intubation occurred within an average of 3.27 ± 0.78 min when the KD combination was administered intravenously. This could be attributed to the simultaneous infusion of both ketamine and dexmedetomidine.

In one study on dogs, KP anaesthesia at a 1:1 ratio, premedicated with acepromazine, resulted in intubation in 2.14 ± 0.11 min with ketamine at 2.1 ± 0.22 mg kg^−1^ and propofol at the same dose (Imani et al. 2016). In the current study, no premedication was administered, and intubation was achieved in 2.73 ± 0.88 min using ketamine at 1.93 ± 0.31 mg kg^−1^ and propofol at 3.87 ± 0.62 mg kg^−1^. Acepromazine may have contributed to the shorter duration compared to KP alone.

Although the intubation times were similar, the effects of the KP and KD combinations on the cardiopulmonary system are different. The MAP was increased by up to 42% in the first 15 min of KD anaesthesia. Upon rapid intravenous injection of dexmedetomidine, it was observed that blood pressure initially exhibited a brief period of hypertension, followed by a decrease. This is probably due to the biphasic response of blood pressure to dexmedetomidine, which is mediated by its α2 adrenergic properties (Pan et al. 2021; Philipp et al. 2002). The administration of KP in dogs did not induce any alterations in MAP (Henao‐Guerrero and Riccó 2014). Compared to propofol alone (Martinez‐Taboada and Leece 2014), the combination of ketamine and propofol maintained MAP in this study. The results of this study indicate that the combined administration of ketamine and propofol had a neutralizing effect on each other, leading to an insignificant impact on MAP. In contrast, MAP was a noteworthy elevation in the KD combination. Therefore, we assume that this increase resulted from the KD combination having a more robust impact on raising systemic vascular resistance than the KP combination.

The intramuscular administration of dexmedetomidine in dogs significantly reduces HR during the initial 15 min. This decrease can be as much as 55%, attributed to the reduction in sympathetic outflow caused by dexmedetomidine (Congdon et al. 2011; Gertler et al. 2001). However, in this study, dexmedetomidine was combined with ketamine and administered intravenously as a single bolus, and it only resulted in a 13% reduction in HR. This modest reduction may be explained by ketamine's sympathomimetic properties, which can enhance catecholamine and dopamine activity in the cardiac system (White and Ryan 1996).

The administration of ketamine in combination with propofol has demonstrated a consistent reduction in HR when administered after premedication (Lerche et al. 2000). However, in the present study, the KP combination was administered without premedication, resulting in a gradual improvement of HR without initially decreasing over time while remaining within clinically acceptable limits. This could be attributed to the strong antinociceptive effect of ketofol combinations in dogs (Ko et al. 2023), which maintain stable cardiac variables.

Studies conducted on dogs have indicated that combining dexmedetomidine and ketamine maintains respiratory variables within acceptable reference ranges (Barletta et al. 2011). In contrast, the KD combination caused significant fluctuations in SpO_2_ and *f*_R_ over time in the present study. Despite ketamine's mitigating effect on propofol‐induced respiratory depression (Green et al. 2015), a significant decrease in SpO_2_ levels was also noted in the KP group compared to the KD group. This potential for SpO_2_ reduction using the KP combination (Martinez‐Taboada and Leece 2014), suggests that manual ventilation support should be considered for dogs anesthetized with the KP combination.

Despite the known hypothermic effect of dexmedetomidine (Granholm et al. 2007), the current study did not detect a significant decrease in body temperature with the administration of dexmedetomidine. We assume that this could be due to dexmedetomidine's inability to suppress the hypothermia‐protective effect of ketamine (Ikeda et al. 2001). However, dogs receiving the KP combination observed a statistically significant decrease in body temperature. This agrees with previous studies on the effect of ketamine combined with propofol on body temperature (Seliškar et al. 2007). This effect is believed to be due to the suppression of hypothalamic activity by general anaesthesia (Armstrong et al. 2005) and the heat loss in the arteriovenous shunts caused by the peripheral vasodilator effect of propofol (Ikeda et al. 2001).

In the literature, ketofol combinations are typically prepared in a 1:1 ratio (Martinez‐Taboada and Leece 2014; Shinde et al. 2018). However, in the present study, a 1:2 ratio was employed. A previous study has recommended using KP at a 1:2 ratio (Şenocak and Yanmaz 2024). Three boluses of KP 1:2 were almost sufficient to maintain anaesthesia for a 30‐min duration. Reducing the second bolus by 30% and the third bolus by 44% compared to the first provided satisfactory anaesthesia during castration surgery. The reduction of the amounts of the boluses over time may be attributed to the accumulation of the propofol (Allegaert et al. 2007).

Rapid and high‐quality recovery is desirable following surgical procedures in dogs. Poor recovery quality is characterized by vocalization, agitation and/or uncoordinated movements, which may pose a risk to surgical wounds (Holton et al. 2001). In the current study, a single intravenous bolus of KD provided a smooth and prolonged recovery, with no withdrawal reflex observed during the surgery. This may be attributed to the long‐lasting effect of dexmedetomidine, which reduces the incidence of poor‐quality recovery in dogs (Hunt et al. 2014). Similarly, the recovery was smooth and without complications in the KP group.

Total intravenous anaesthesia is widely used in human and veterinary medicine, often requiring additional doses for painful procedures. In humans, KP and KD are effective for paediatric patients during painful treatments (Canpolat et al. 2012; Abdalla et al. 2015). KP has shown efficacy across various surgeries without premedication (Friedberg 1999). In veterinary practice, this combination provides potent antinociception and stable cardiac variables in dogs and is suitable for cats undergoing ovariectomy (Ko et al. 2023; Ravasio et al. 2012). In rabbits, successful anaesthesia induction and intubation depend on the dosage (Santos et al. 2016). The current study has demonstrated the efficacy of total intravenous anaesthesia using a combination of ketamine and either propofol or dexmedetomidine in facilitating painful surgical procedures, including orchiectomy. Administration of the ketamine and dexmedetomidine combination required only a single bolus injection, whereas multiple bolus injections were necessary when ketamine was combined with propofol.

This research has certain limitations that must be noted. First, the end‐tidal carbon dioxide (EtCO_2_) levels were not recorded due to the unavailability of the capnography module. It is acknowledged that dexmedetomidine, due to its potent peripheral vasoconstrictive effects, can slow blood flow in the peripheral tissues, thereby prolonging capillary transit time. This mechanism may enhance oxygen extraction and result in the tongue appearing pale blue or grey, which could potentially affect the accuracy of SpO_2_ measurements. Therefore, SpO_2_ readings in the KD group may not accurately reflect arterial oxygenation status. This limitation, combined with the non‐availability of EtCO_2_ monitoring, should be considered when interpreting the findings related to oxygenation and respiratory parameters in this study. Future studies incorporating EtCO_2_ monitoring could provide more reliable assessments of respiratory function and improve the validity of oxygenation measurements under these anaesthetic protocols. Second, the sample size was just calculated for intubation time. This was because the study was conducted as part of a spay/neuter programme at a shelter, and all eligible dogs were enrolled. Third, measuring the MAP through an indirect method is widely recognized as vulnerable to substantial bias when blood pressure levels are elevated.

In conclusion, the present study has shown that both the KP and KD combinations have similar effects on intubation time, surgery time, extubation time, recovery time and quality. They were employed to anaesthetize dogs undergoing castration surgery. KP anaesthesia required at least three intermittent boluses to achieve a 30‐min effect duration. The administration of KD was associated with a persistent decrease in HR and an increase in MAP. In contrast, KP had a comparatively lesser impact on cardiac variables. Dogs undergoing KP anaesthesia are more susceptible to experiencing respiratory depression in comparison to those receiving the KD combination. KP was observed to be more likely to cause a decrease in body temperature than the KD combination. KP was deemed more manageable than KD due to its shorter duration and lack of α2‐mediated cardiovascular suppression. However, it is essential to maintain body temperature and provide oxygen support when administering the KP combination. A rise exceeding 20% in two of HR, MAP or *f*_R_ is a good indicator for starting bolus infusion. The interval between two boluses is approximately 8–9 min.

## Author Contributions

**Mumin Gokhan Senocak**: conceptualization, investigation, writing – original draft, methodology, validation, visualization, data curation.

## Ethics Statement

This prospective study was approved by the Atatürk University Local Board of Ethics Committee (2022/6‐99 and 2022/10‐198).

## Conflicts of Interest

The author declares no conflicts of interest.

### Peer Review

The peer review history for this article is available at https://www.webofscience.com/api/gateway/wos/peer‐review/10.1002/vms3.70412.

## Data Availability

The data that assist the study results are available from the corresponding author upon reasonable request.
